# Case Report: A Highly Variable Clinical and Immunological Presentation of IKAROS Deficiency in a Single Family

**DOI:** 10.3389/fimmu.2022.865838

**Published:** 2022-04-11

**Authors:** Taco W. Kuijpers, Samantha A. M. Tromp, Ester M. M. van Leeuwen, Godelieve J. de Bree

**Affiliations:** ^1^Department of Pediatric Immunology, Rheumatology and Infectious Diseases, Emma Children’s Hospital, Amsterdam University Medical Center, University of Amsterdam, Amsterdam, Netherlands; ^2^Department of Blood Cell Research, Sanquin Research and Landsteiner Laboratory of Immunology, Amsterdam University Medical Center, University of Amsterdam, Amsterdam, Netherlands; ^3^Department of Experimental Immunology, Amsterdam Institute for Infection and Immunity, Amsterdam University Medical Center, University of Amsterdam, Amsterdam, Netherlands; ^4^Department of Internal Medicine, Institute for Infection and Immunity, Amsterdam University Medical Center, University of Amsterdam, Amsterdam, Netherlands

**Keywords:** Ikaros, IKZF1, inborn error of immunity (IEI), common variable immunodeficiency (CVID), whole exome sequencing (WES)

## Abstract

Here we describe a novel mutation in the *IKZF* gene encoding IKAROS, as the cause of common variable immunodeficiency (CVID). The identification of the same defect in the *IKZF* gene with manifestations of asymptomatic selective IgA deficiency and chronic ITP in the father and her younger brother, respectively, demonstrates the large variability of this genetic defect in one single family, while living in the same environment with a relatively similar genetic background. As discussed, clinical penetrance of the molecular defects identified by mutations in *IKZF* and other common gene defects in CVID in familial immune-related abnormalities makes genetic testing a necessary step for diagnosis, management, and counseling, as part of the routine immunological workup.

## Introduction

Humoral immunodeficiencies are the most common immune disorders in children and adults and are characterized by the occurrence of infections and/or autoimmune manifestations (1). Nowadays, the term “inborn errors of immunity” (IEI) is more commonly used (2), since the clinical presentation often consists of overlap of immunodeficiency, immune dysregulation, autoinflammation, and autoimmunity. In recent years, the number of mono-genetic defects underlying primary immunodeficiencies (PIDs) has increased exponentially (3). The number of genes involved in IEI is already over 450 to date. With the concomitant increase of genetic testing as part of the diagnostic workup, we are gaining better insight into the clinical presentation of immune disorders and new avenues for treatment and clinical follow-up are opened. Timely recognition of an immune disorder is important to limit organ damage from severe or recurrent infections and treat autoimmune or inflammatory manifestations. At the same time, there are emerging data that show that the clinical picture of IEI that is explained by monogenetic defects can be highly variable. In this article, we illustrate the variable clinical picture and immunological phenotype within one single family with a humoral immunodeficiency caused by the same mutation in IKAROS.

## Materials and Methods

### Patients

The index and her sib were diagnosed and treated at the Amsterdam University Medical Center, Netherlands, where they received treatment according to established clinical guidelines. All blood samples used for immunobiological analyses were obtained after informed consent (National PID study: NL40331.078).

### Peripheral Mononuclear Cell Isolation

PBMCs were isolated by density centrifugation over Ficoll-Paque media (4).

### Immunophenotyping

Absolute numbers of thrombocytes and ferritin levels were routinely measured according to established diagnostic guidelines. Absolute numbers of lymphocytes were determined with Multitest 6-color reagents (BD Biosciences, San Jose, USA), according to the manufacturer’s instructions. For additional flow cytometry, PBMCs were resuspended in PBS, containing 0.5% (w/v) BSA and 0.01% sodium azide and incubated with saturating concentrations of fluorescently labeled conjugated monoclonal antibodies. Patient samples were analyzed simultaneously with PBMCs from healthy donors. The following directly conjugated monoclonal antibodies were used: CD3-APC, CD3-APC-H7, CD4-PE-Cy7, CD4-PerCP-Cy5.5, CD8-PerCP-Cy5.5, CD19-PerCP-Cy5.5, CD20-APC, and CD56-FITC from BD Biosciences (San Jose, USA); IgD-PE and gamma-1 isotype from BD Pharmingen (San Diego, USA); CD20-APC from BioLegend (San Diego, USA); CD16-FITC, and CD2-FITC from Sanquin (Amsterdam, Netherlands); and CD45RA-PE (RD-1) from Beckman Coulter (Brea, USA). Unconjugated antibodies against SAP [H00004068-M0] from Tebu-Bio (Heerhugowaard, Netherlands) were fluorescently labeled with PE using the Lightning-Link RPE Conjugation Kit [703-0030] from Innova Biosciences (Cambridge, UK). Analysis of cells was performed using a FACSCanto II flow cytometer and FlowJo software.

### B-Cell and T-Cell Activation *In Vitro*

To analyze the *in vitro* activation of B and T cells, PBMCs were resuspended in PBS at a concentration of 5 × 10^6^–10 × 10^6^ cells/ml and labeled with 0.5 µM carboxyfluorescein succinimidyl ester (CFSE) (Molecular Probes, Thermo Fisher Scientific) in PBS for 10 min at 37°C under constant agitation. Cells were washed and subsequently resuspended in Iscove’s Modified Dulbecco’s medium (IMDM) supplemented with 10% fetal calf serum (FCS, BioWhittaker), antibiotics, and 3.57 × 10–4% (v/v) β-mercaptoethanol (Merck). Labeled PBMCs were plated containing a fixed number of 10 × 10^4^ B cells per well and were cultured in a 48-well flat-bottomed plate for 6 days at 37°C. Cells were stimulated with saturating amounts of anti-IgM mAb (clone MH15; Sanquin, Amsterdam, Netherlands), anti-CD40 mAb (clone 14G7; Sanquin), and 20 ng/ml IL-21 (Invitrogen, Waltham, MA, USA), or 1 µg/ml CpG oligodeoxynucleotide 2006 (Invivogen, San Diego, CA, USA) and 100 U/ml IL-2 (R&D Systems, Minneapolis, MN, USA). For T-cell stimulation, saturating amounts of anti-CD3 (clone 1XE) and anti-CD28 (clone 15E8) were added. Proliferation of B and T cells was assessed by measuring CFSE dilution in combination with the same mAbs used for immunophenotyping. Cells were analyzed with a FACSCanto II flow cytometer and FlowJo software.

### Immunoglobulin Production *In Vitro*

The secretion of immunoglobulins by mature B cells was assessed by testing culture supernatant for secreted IgM and IgG with an in-house ELISA using polyclonal rabbit anti-human IgM and IgG reagents and a human serum protein calibrator all from Dako (Glostrup, Denmark), as described previously (4).

### Sequencing

Whole-exome capture and sequencing were performed using SeqCap EZ MedExome (Roche NimbleGen, Pleasanton, CA, USA). The resulting libraries were sequenced on a HiSeq4000 (Illumina, San Diego, CA, USA) according to the manufacturer’s recommendations for paired-end 150-bp reads. Alignment of sequence reads to the human reference genome (hg19) was done using BWAMEM 0.7.10 (bio-bwa.sourceforge.net/), and variants were called using the GATK3.3 software package (www.broadinstitute.org/gatk/). Filtering of variants was done using Alissa Interpret (Agilent Technologies, Santa Clara, CA, USA). Variants with <5 reads, a frequency of more than 1% in public (ESP, dbSNP, 1KG), and/or in-house databases were excluded. *De novo*, homozygous, or compound heterozygous variants present in exons or within +/- 6 nt in the intron were evaluated (5). Sequencing had been independently confirmed by a whole genome sequencing approach (6).

## Results

### Case Description

The index was a female teenager referred by the general practitioner to the pediatrician at the age of 14 with fatigue complaints. Physical examination and general laboratory analysis were unremarkable. Her periods were regular, and there were no cardiac or respiratory abnormalities. A year later, she was again referred by the general practitioner to a consultant of pediatric infectious diseases and clinical immunology after three pneumonias with fever and cough complaints, all of which responded well to antibiotics. All sputum cultures remained negative. Blood tests yielded normal results for sedimentation, CRP, blood count and differentiation, and liver and kidney function. There was a normal TSH value. The IgA was absent (<0.04 g/l), the IgG (4.6 g/l) was low, and the IgM (0.31 g/l) was low–normal. EBV and CMV serology were IgG negative. Vaccination responses against the 23-valent pneumococcal polysaccharide (PPS) vaccine and the protein component tetanus toxoid (TetTox) were determined, and both vaccination responses were reduced (**Table 1**). There was no IgG detectable against live-attenuated measles–mumps–rubella (MMR) despite previous immunization according to our national vaccination program at 14 months and 9 years of age. She had previously experienced chickenpox without clinical problems, with a positive VZV IgG serology. Within 6 months following the diagnostic vaccination studies, antibody levels dropped to almost undetectable levels: IgG 1.7 g/l and IgM 0.04 g/l, in the presence of normal CD4^+^ and CD8^+^ T-cell numbers and NK-cell numbers, but a markedly reduced B-cell number. Taken together, following the pneumonias, hypogammaglobulinemia, and low response on pneumococcal vaccination, a definite diagnosis of common variable immunodeficiency (CVID) was made (www.ESID.org; **Table 2**). Treatment with prophylactic antibiotics and immunoglobulin supplementation therapy (IVIG) was initiated with an IgG trough value above 6.0 g/l. Since that moment, the patient has been without infections.

**Table 1:** Hematology and immunology parameters in index, sib and father at presentation.

Laboratory results	Index	Sib	Father	Normal ranges	
	*14 years old*	*12 years old*	*47 years old*		
Hematology				*Adolescent*	*Adult*
Hemoglobin (mmol/L)	8.2	6.6	8.4	6.5–10	8.5–10.5
Platelets (10^9^/L)	181	32	160	150–450	150–400
Leukocytes (10^9^/L)	5.9	3.7	4.3	4–14	4–10.5
Lymphocytes (total, 10^9^/L)	2.90	1.53	1.14	1–5	1.5–4
Monocytes (10^9^/L)	0.57	0.66	0.57	0.1–1	0.1–1
Neutrophils (10^9^/L)	2.28	1.32	2.42	1.5–8	1.8–7.2
Eosinophils (10^9^/L)	0.14	0.19	0.12	0–0.5	0–0.5
**Lymphocyte counts**					
CD19^+^ B cells (10^9^/L)	0.086	0.167	0.106	0.1–0.6	0.1–0.6
CD3^+^ T cells (10^9^/L)	1.693	1.305	1.028	0.7–3.5	0.7–3.5
CD3^+^CD4^+^ T cells (10^9^/L)	0.578	0.639	0.457	0.3–2.1	0.3–2.1
CD3^+^CD8^+^ T cells (10^9^/L)	0.924	0.602	0.526	0.2–1.2	0.2–1.2
CD4/CD8 ratio	0.6	1.1	0.9	0.9–3.6	0.9–3.6
CD3^-^CD16^+^CD56^+^ NK cells (10^9^/L)	0.120	0.204	0.205	0.07–1.2	0.07–1.2
**Immunology**					
IgG (g/L)	4.6	7.8	7.2	4.1–12.3	7–16
IgA (g/L)	<0.04	0.08	<0.04	0.14–2.6	0.7–4
IgM (g/L)	0.31	0.54	2.94	0.23–2	0.4–2.3
**Vaccine response*^a^***					
Anti-PPS23 (ratio of 23 serotypes)	2.32*^b^*	NA	NA	>2.0	>2.0
	(2.59>6.0)				
Anti-Tet-Toxoid (ratio)	3.33*^b^*	NA	NA	>2.0	>2.0
	(0.03>0.1)				
**Other**					
ANA	Negative	Negative	Negative		
CMV IgG	Negative	Negative	Negative		
EBV IgG (viral capsid antigen)	Negative	Negative	Positive		
CRP (mg/L)	0.8	<0.3	NA	0–5	0–5
ASAT (U/L)	20	25	NA	0–40	0–40
ALAT (U/L)	15	15	NA	0–45	0–45
Albumin (g/L)	48	47	NA	37–55	35–50
Creatinine (µmol/L)	42	44	NA	35–100	75–110
FT4 (pmol/L)	10.9	16.3	NA	10–23	12–22
TSH (mE/L)	7.3	2.3	NA	0.5–5	0.5–5

^a^Data were taken at day 0 pre-vaccination and at day 35 post-vaccination for comparison and divided to calculate a ratio. The ratio should be above 2.0, taking into account the concentration of anti-pneumococcal antibodies or anti-tetanus toxoid antibodies at start (when compared to immunized controls).

^b^No/little increase in IgG titer for both pneumococcal polysaccharide and tetanus toxoid after vaccination. HD show an evident increase in titer after vaccination.

NA, not assessed.

**Table 2:** Definition of CVID.

Definition of common variable immunodeficiency (CVID) according to the criteria of the European Association for Immunodeficiencies (ESID; www.esid.org).
1. Recurrent infections, autoimmunity, lymphoproliferative disease, or granulomatous abnormalities
2. Low serum IgG, in combination with a low IgM and/or IgA
3. Impaired vaccination response and/or low number of IgA- and IgG-positive memory B cells

Given the relatively young age of the index patient in combination with the described (“uitgesproken”) clinical and immunological abnormalities, the family history was investigated. The parents of the index patient were unrelated. The father once had an Ig spectrum determined during screening of vaccination responses because of his frequent professional travels to tropical countries. He had a normal IgG and IgM but a complete IgA deficiency. He was without any complaints at all. His T, B, and NK cells were within the normal range (**Table 1**). The mother of the index patient was healthy, and no immunological abnormalities were observed. The younger brother of the index patient had presented to our hospital 6 months before the referral of the index patient herself. He had a whooping cough and a mild thrombopenia upon a blood test. Antiplatelet autoantibodies were present, along with selective IgA deficiency. He was treated with azithromycin, and the cough slowly cleared over the following 2–3 months. However, during this follow-up, we diagnosed chronic idiopathic thrombopenia (cITP) because of persistent low platelet counts around 50 × 10^9^/l—without any clinical bleeding tendency though, and normal vaccine reactivity against pneumococcal vaccine, tetanus vaccine, and measles–mumps–rubella vaccine.

### Genetic Analysis and Immunophenotype

**Figure 1:**
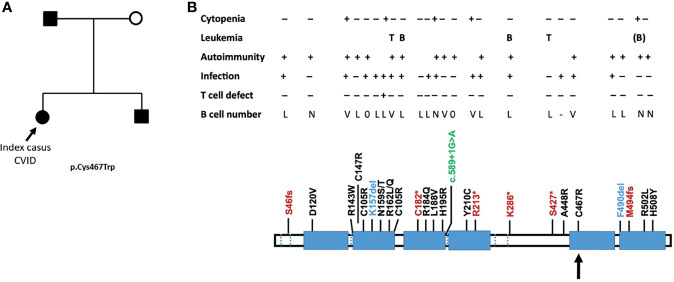
**(A)** Family tree with the genetically affected members following haploinsufficiency with variable penetrance. The index case is indicated by an arrow, who presented with a typical CVID. **(B)** The position of the mutation is with an arrow (adapted from Yamashita et al. (7). Mutations comprise early stop codons (red), missense mutations (black), a deletion (blue), and splice defect (green). Larger deletions causing a complete haploinsufficiency have also been described (7). L, low; V, variable; N, normal.

**Figure 2:**
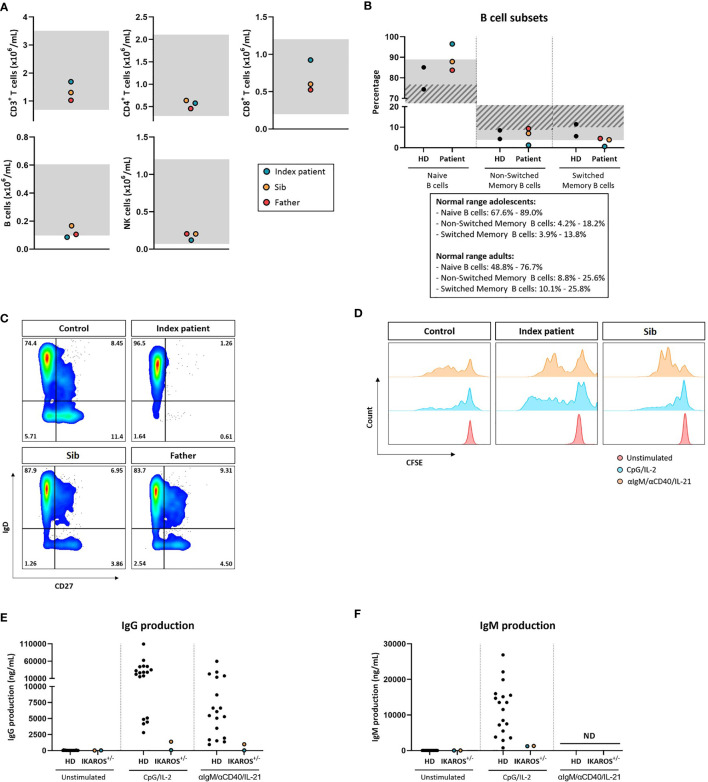
**(A)** Absolute B-cell, T-cell, and NK-cell numbers. Gray indicates age-dependent normal values. **(B)** Percentages of naive (CD27^-^IgD^+^), non-switched memory (CD27^+^IgD^+^), and switched memory B cells (CD27^+^IgD^-^) within the total B-cell pool (CD19^+^CD20^+^). Gray indicates normal values for adolescents; gray stripe indicates normal values for adults. **(C)** Representative flow cytometry plots of B-cell subsets. **(D)** Proliferation of B cells measured by CFSE dilution. **(E)** IgG production *in vitro* after 20 days of culture in ng/mL measured by ELISA. **(F)** IgM production *in vitro* after 20 days of culture in ng/mL measured by ELISA.

The family history with 3 family members affected prompted us to perform a next-generation-sequencing (NGS) panel of 419 genes in all four members (parents, index, and sib) of the family. A pathogenic mutation in the *IKZF1* gene encoding the protein IKAROS was identified in the index case. The mutation was a heterozygous mutation in c.1401C>G (p.Cys467Trp) in the zinc finger 5 domain (ZF5) (**Figure 1A**). The same pathogenic mutation was found to be present in the father and her younger brother, being absent in the mother (**Figure 1B**). Structurally, IKZF proteins share N-terminal (ZF5) zinc-finger domains that mediate DNA binding and two C-terminal zinc-finger domains for multimerization. IKZF proteins can form both homodimers and heterodimers and are a component of the nucleosome remodeling and histone deacetylase complex and polycomb repressive complex 2 (PRC2) (8). Mutations at the same location in the ZF5 domain (c.1401) have been described recently and were shown to be missense mutations (9). These mutations in the dimerization domain result in full or partial loss of homo- and heterodimerization of IKAROS and other transcription factors such as Helios and AIOLOS (10). IKAROS is a transcription factor that is important for the development of B lymphocytes and other cells of the immune system including T cells and dendritic cells (8). Immunophenotyping showed normal absolute numbers of T and NK cells, but a reduced absolute count of B cells in the index. Absolute lymphocyte counts (B, T and NK cells) were normal in her sib and father (**Figure 2A**). Phenotypical analysis in the index patient and her sib showed very few memory B cells. Phenotypic analysis of the father showed a normal distribution of B-cell subsets (**Figures 2B, C****)**. Distribution of CD4^+^ and CD8^+^ T-cell subsets was normal in the index patient, her sib, and the father (**Supplemental Figure 1A**). In addition, a B- and T-cell proliferation assay was performed to investigate development of plasmablasts and *in vitro* IgG and IgM production on various conditions (**Figures 2D–F**). The proliferation assay showed normal expansion of B and T cells in the index patient and her sib (**Figure 2D**). The *in vitro* production of IgG and IgM in the supernatant was impaired as compared to healthy controls (**Figures 2E, F****)**. Finally, since the index patient and her sib had undetectable IgA serum levels, we analyzed the membrane IgA expression on the B cells (**Supplemental Figure 1B**). This showed a lack of IgA surface expression on the memory B cells that is suggestive for a B-cell maturation defect toward IgA positivity.

## Discussion

In the present paper, we describe a family in which 3 family members were diagnosed with the same IKAROS mutation but have a very diverse clinical picture.

CVID is a heterogeneous clinical picture characterized by late-onset hypogammaglobulinemia in the absence of predisposing factors and a polygenic background with a considerable risk to develop autoimmunity, granulomatous inflammation, and an undeniable predisposition to malignancy (11, 12). In 10% of cases, the disease may have a monogenic hereditary component (4, 13). Most CVID patients have normal numbers of B cells but lack plasma cells and are variable in their clinical presentation within one and the same pedigree with CVID for the most frequently identified gene defects in NFKB1, CTLA4, and PIK3CD (4, 14, 15), as now also exemplified in our and other kindreds with an *IKZF1* defect and low B-cell numbers (16).

While somatic *IKZF1* alterations were known to be involved in the pathogenesis of leukemia in human subjects (17), over the last years various immunodeficiency syndromes caused by germline *IKZF1* mutations have been described. Depending on the location of the mutation in the gene, this autosomal-dominant inheritable immune disorder can range from severe to mild (18, 19). The mutation in our family (p.Cys467Trp) is located in exon 8 (zinc finger domain 5) at a known mutation position (p.Cys467Arg) (9). Most IKAROS mutations result in a greatly increased susceptibility to infectious diseases, whether or not in combination with autoimmune diseases. Sometimes autoimmune diseases, such as ITP, SLE, and IBD are in the foreground (7, 20–22), while in others, these germline mutations can give rise to B- or T-cell leukemia (7, 17, 18, 23), in which an absolute predictive parameter or genotype–phenotype relationship is still elusive.

Mutations in the *IKZF1* gene do not have to be limited to a humoral immune disorder in which only the B cells are affected but can become clinically overt as combined T- and B-cell disorder with *Pneumocystis jirovecii*, bacterial, and viral infections associated with a limited number of specific mutations in the gene (19). In the latter case, the number of T cells may not be abnormal and only more in-depth immunophenotyping (detailed subset analysis of naive, memory, and effector T cells) and cellular function tests are required to determine the impact of the IKAROS-associated T-cell defect. These children are recognized early (24) and should undergo a hematopoietic stem cell transplant (HSCT) to prevent further serious infections and progressive organ damage (24, 25).

Although a genetic diagnosis can only be made in a small percentage of CVID patients, an analysis of the entire “exome” (all coding parts, i.e., 2%, of the genome) or the entire “genome” (to interpret promoter regions, splice-site and intron regions, and assumed intergenic super-enhancer regions can be a powerful means of making an explanatory genetic diagnosis, especially in familial cases (5, 6). On the basis of genetic findings, additional immune research can be carried out in specialized laboratories to map the immune defect in a more targeted manner. The major challenge nowadays is to produce the evidence of the pathogenic role of a novel gene defect or to demonstrate that variants within a known gene explain the disease if co-segregation is not sufficient to indicate the pathogenicity of a given gene variant.

A genetic diagnosis is important to give patient-oriented advice about the risk of infections, autoimmunity, inflammation, or predisposition to cancer, supported with a disease-tailored monitoring approach. A genetic diagnosis may indicate increased sensitivity to X-rays or radioactivity in so-called DNA repair defects (26). Such knowledge is important to prefer certain imaging (ultrasound and MRI instead of X-ray examination or PET scanning). The genetic nature of a disease may also indicate the expected progressive course of a disease, for which a donor search must be initiated for HSCT; prenatal diagnostics can be performed or pedigree-based genotyping can be offered to pick up additional, genetically affected members.

The identification of the same defect in IKAROS with a variety of manifestations from asymptomatic IgA deficiency, chronic ITP, and CVID in a small single family demonstrates that living in the same environment with a relatively similar genetic background may still result in highly variable manifestations among family members which may be explained by differences in the epigenetic modifications, which also in CVID in identical twins have been indicated to exist as a next avenue to understand clinical penetrance in genetically affected family members (27, 28). In our family, all three family members appeared to be affected and the mother not. The family was informed of the genetic finding and the inheritance for future prenatal testing if requested. The mutation in our patient could potentially perturb dimerization with itself or with other members of the IKZF family including HELIOS (*IKZF2*), AIOLOS (*IKZF3*), EOS (*IKZF4*), and PEGASUS (*IKZF5*) (8). When asked whether there is an increased risk of developing leukemia, we could only indicate that the risk of malignant transformation does not seem to be high but cannot be denied, being recently reported in a series with increased risk of autoimmunity and malignancy (9).

In summary, the present case report illustrates the diverse clinical phenotypes that can accompany a gene defect in a single family. These data underscore the merit of genetic analysis when patients within a family present with different infectious and autoimmune manifestations.

## Data Availability Statement

The datasets presented in this study can be found in online repositories. The names of the repository/repositories and accession number(s) can be found as follows: https://www.ncbi.nlm.nih.gov/snp/rs1585040113?horizontal_tab=true#variant_details; https://www.ncbi.nlm.nih.gov/clinvar/variation/827708/?new_evidence=false.

## Ethics Statement

The studies involving human participants were reviewed and approved by Dr. V.A.S.H. Dalm from the Department of Internal Medicine, Division of Clinical Immunology and Department of Immunology, Erasmus University Medical Center, Rotterdam, the Netherlands (national PID study: NL40331.078). Written informed consent to participate in this study was provided by the participants’ legal guardian/next of kin. 

## Author Contributions

TK wrote the paper. TK and GB were responsible for the clinical care and clinical treatment. ST was responsible for the final analysis, generated the figures, and wrote part of the text. EL performed the immunological measurements and together with GB critically reviewed the manuscript. All authors contributed to the article and approved the submitted version.

## Funding

This report was funded by the Center of Immunodeficiencies Amsterdam (CIDA grant 2015).

## Conflict of Interest

The authors declare that the research was conducted in the absence of any commercial or financial relationships that could be construed as a potential conflict of interest.

## Publisher’s Note

All claims expressed in this article are solely those of the authors and do not necessarily represent those of their affiliated organizations, or those of the publisher, the editors and the reviewers. Any product that may be evaluated in this article, or claim that may be made by its manufacturer, is not guaranteed or endorsed by the publisher.

## Supplementary Material

Supplementary Figure 1 **(A)** Percentages of naive (CD27^+^CD45RA^+^), memory (CD27^+^CD45RA^-^) and effector memory T cells (CD27^-^CD45RA^+^) within total CD4^+^ and CD8^+^ T cell pool. **(B)** Surface IgM, IgG and IgA expression ex vivo in the index patient and sibling.
