# Supplementary figures and images for: Case Report: A Highly Variable Clinical and Immunological Presentation of IKAROS Deficiency in a Single Family
Source: Front Immunol. 2022 Apr 11;13:865838. doi: 10.3389/fimmu.2022.865838 (PMC9036438; doi:10.3389/fimmu.2022.865838)

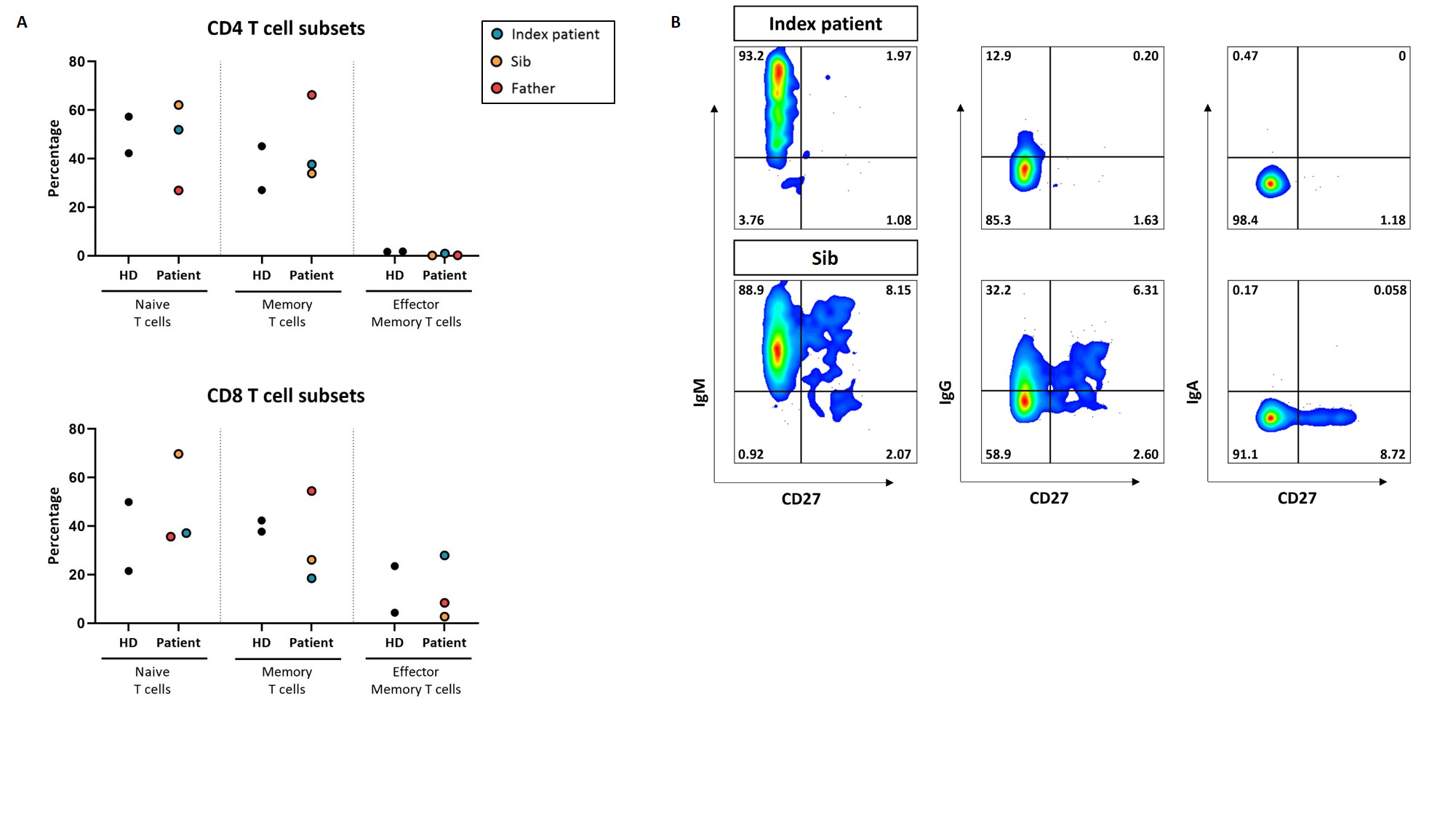

Supplement: Supplementary Figure 1 — (A) Percentages of naive (CD27+CD45RA+), memory (CD27+CD45RA-) and effector memory T cells (CD27-CD45RA+) within total CD4+ and CD8+ T cell pool. (B) Surface IgM, IgG and IgA expression ex vivo in the index patient and sibling. [file Image_1.jpeg]
